# *In-situ*-Investigation of Enzyme Immobilization on Polymer Brushes

**DOI:** 10.3389/fchem.2019.00101

**Published:** 2019-03-07

**Authors:** Meike Koenig, Ulla König, Klaus-Jochen Eichhorn, Martin Müller, Manfred Stamm, Petra Uhlmann

**Affiliations:** ^1^Department of Nanostructured Materials, Leibniz Institute of Polymer Research Dresden, Dresden, Germany; ^2^Department of Analytics, Leibniz Institute of Polymer Research Dresden, Dresden, Germany; ^3^Department of Polyelectrolytes and Dispersions, Leibniz Institute of Polymer Research Dresden, Dresden, Germany; ^4^Physical Chemistry of Polymer Materials, Technische Universität Dresden, Dresden, Germany; ^5^Department of Chemistry, University of Nebraska-Lincoln, Lincoln, NE, United States

**Keywords:** polymer brushes, enzymes, enzyme immobilization, responsive coatings, quartz-crystal microbalance, ellipsometry

## Abstract

Herein, we report on the use of a combined setup of quartz-crystal microbalance, with dissipation monitoring and spectroscopic ellipsometry, to comprehensively investigate the covalent immobilization of an enzyme to a polymer layer. All steps of the covalent reaction of the model enzyme glucose oxidase with the poly(acrylic acid) brush by carbodiimide chemistry, were monitored *in-situ*. Data were analyzed using optical and viscoelastic modeling. A nearly complete collapse of the polymer chains was found upon activation of the carboxylic acid groups with N-(3-Dimethylaminopropyl)-N′-ethylcarbodiimide and N-Hydroxysuccinimide. The reaction with the amine groups of the enzyme occurs simultaneously with re-hydration of the polymer layer. Significantly more enzyme was immobilized on the surface compared to physical adsorption at similar conditions, at the same pH. It was found that the pH responsive swelling behavior was almost not affected by the presence of the enzyme.

## 1. Introduction

The immobilization of enzymes on solid supports has been an active field of research for several decades, due to its impact on industrial applications such as biocatalysts, or in sensor technologies (Rosevear, 1984; Zoungrana et al., 1997; Tischer and Wedekind, 1999; Sheldon and van Pelt, 2013; Zdarta et al., 2018). In these applications, the binding to macroscopic surfaces ensures efficient usage, since separation from the product and re-use is facilitated, compared to the soluble form of the enzyme. Additionally, immobilization often results in enhanced thermal and operational stability. In general, there are three types of immobilization techniques: binding to a pre-formed carrier or support, encapsulation within a carrier matrix, and cross-linking of enzymes. Binding to a support can be done by adsorption via non-covalent or ionic bonds, or by chemical coupling. The immobilization might change the activity of enzymes, either intrinsically, by a change in the conformation or mobility of the enzyme, or by a modification of the accessibility of the enzyme. These changes mostly lead to a decrease in the activity of the immobilized enzyme, whereas in some cases also an increased activity, as compared to the free enzyme in the solution, was observed (Rodrigues et al., 2013; Wu et al., 2014; Zhang et al., 2015).

Although physical adsorption, for instance via ionic interactions, is a very common method to immobilize enzymes on supports, one prominent problem associated with physical adsorption of enzymes on carriers is the rather poor stability of these interactions (Hanefeld et al., 2009; Jesionowski et al., 2014; Koenig et al., 2016). The so called “initial burst” and leaching of the biocatalyst into the surrounding medium, causes severe problems for industrial applications. This disadvantage can be overcome by covalent coupling of the enzyme to the carrier (Rosevear, 1984; Hanefeld et al., 2009). Additionally, this can lead to increased thermal and chemical stability of the enzyme, due to the formation of multiple bonds with the support. On the downside, the immobilization process is more complex, since modification steps for the support or the enzyme are necessary and irreversible inactivation of the enzyme, by the formation of the covalent bond, has to be averted.

Polymer brushes, as soft and flexible carriers with tailored properties and a high number of functional groups, have emerged as suitable candidates to act as an immobilizing layer to equip various support materials with enzymes (Lane et al., 2011; Yuan et al., 2011; Feng et al., 2014; Bayramoglu et al., 2017). Stimuli-responsive biocatalytic materials can be designed using responsive polymer brushes. These brushes are capable of reacting to external stimuli, generally by reversible swelling-deswelling behavior (Ito et al., 1989; Bocharova et al., 2010; Crulhas et al., 2014; Dbner et al., 2017). In previous studies of our group, poly (acrylic acid) brushes in particular emerged as a versatile polymer that can efficiently bind biomolecules and has stimuli-responsive properties at the same time (Bittrich et al., 2010; Psarra et al., 2015, 2017; Koenig et al., 2016; König et al., 2018).

Various methods can be applied to characterize the enzyme immobilized on the polymer brush. For qualitative characterization, infrared spectroscopy and x-ray photoelectron spectroscopy are the most frequently used techniques. Colorimetric assays are mostly applied for quantitative detection of the immobilized enzyme, such as the assay after Bradford (Xu et al., 2005; Costantini et al., 2013; Zhu et al., 2014). Ellipsometry and quarz crystal microbalance with dissipation monitoring (QCM-D) can also be used Cullen et al. (2008); Ren et al. (2014). Almost exclusively, characterization is done *ex-situ*, to characterize changes on the surface before and after the immobilization step. Direct characterization of the immobilization process *in-situ* has rarely been reported, although a detailed understanding of the ongoing process is crucial for the optimization of the enzyme product (Draghici et al., 2014).

For this report, we used a combined setup of QCM-D and Spectroscopic Ellipsometry (SE) to characterize and quantify the covalent immobilization of Glucose Oxidase (GOx) on poly (acrylic acid) (PAA) brushes, by carbodiimide chemistry *in-situ*. Using viscoelastic and optical modeling, this setup allows a comprehensive study of the thickness, composition, optical, and mechanical properties of the polymer layer and the enzyme (Bittrich et al., 2010; Rodenhausen and Schubert, 2011; Adam et al., 2017). The combined setup ensures the direct comparability of the two techniques.

## 2. Experimental Details

### 2.1. Preparation of Polymer Brushes

PAA Brushes were prepared via an already optimized grafting-to method (Iyer et al., 2003). Like substrates, silica-coated quartz crystals with an approximately 50 nm thick SiO 2 layer (QSX 303, Biolin Scientific, Sweden) were used. Samples were cleaned with ethanol abs. (VWR, Germany) and dried with nitrogen gas. Samples were then activated by oxygen plasma for 1 min at 100 W (440-G Plasma System, Technics Plasma GmbH, Germany). A macromolecular anchoring layer was spin-coated onto the surface from a 0.02 wt.-% solution of poly (glycidyl methacrylate) (PGMA, *M*_*n*_=17 500 g mol^−1^, *M*_*w*_/*M*_*n*_=1.12, Polymer Source, Inc., Canada) in chloroform (Sigma-Aldrich, Germany) and annealed in a vacuum for 20 min at 100 °C to chemically bind the polymer to the activated SiO2 surface. The PAA Guiselin brush layer was grafted by the spin-coating of a 1 wt.-% PAA solution (*M*_*n*_= 26 500 g/mol, *M*_*w*_/*M*_*n*_=1.7, Polymer Source, Inc., Canada) in ethanol, followed by annealing in a vacuum at 80 °C for 30 min and the extraction of the non-covalently bound polymer in ethanol.

### 2.2. Combined QCM-D/SE

The combined QCM-D/SE setup consists of an E1 QCM-D module (Biolin Scientific, Sweden), mounted onto the sample stage of an alpha-SE spectroscopic ellipsometer (J.A. Woollam Co., United States) with a fixed angle of incidence of 65°. A flow rate of 0.1 ml min^−1^ was used for the exchange of liquid in the cell. Experiments were done in 0.01 M sodium phosphate buffer solution (prepared using NaH 2PO 4·2H 2O and Na2HPO4·2H2O) at 22 °C. The sample was first rinsed in pH 7.4 and in pH 6 buffer solution, before a solution of 0.01 M N-(3-Dimethylaminopropyl)-N'-ethylcarbodiimide (EDC) and 0.025 M N-Hydroxysuccinimide (NHS) in pH 6 was introduced. For reaction with the enzyme, a solution of 1 mg ml^−1^ GOx (GOx Type II, G6125 from *Asperigillus Niger*) in pH 6 buffer was applied. Desorption of a non-covalently bound enzyme was done through rinsing steps with pH 6, pH 7.4 and again with a pH 6 buffer solution. All chemicals were purchased from Sigma Aldrich, Germany and used as received.

### 2.3. Ellipsometry Modeling

A simple multi-layer box-model was used to model the optical properties and the thickness of the thin polymer films with and without enzymes from the measured ellipsometric angles Δ and tan(Ψ). This model, which assumes sharp interfaces between all layers as well as a homogeneous distribution of polymer segments and adsorbed enzymes within the polymer layer, has previously been found to sufficiently describe similar thin films (Bittrich et al., 2012; Rauch et al., 2012).

The refractive index (n) and absorption coefficient (k) of the blank silica coated quartz sensors were parameterized by basis-spline (B-spline) functions (Johs and Hale, 2008). The modification of the substrate with polymer layers in the dry state (ambient air conditions with relative humidity ≈40% and 21 °C) was modeled with fixed refractive indices (*n*_*PGMA*_=1.525, *n*_*PAA*_=1.522, determined by measurements of a thick polymer layer for λ = 631.5 nm). Modeling of *in-situ* measurements of the polymer brush layer, activation by EDC/NHS and quantification of the covalently bound enzyme was done according to a previously published method (Koenig et al., 2016). Briefly, a two-parameter Cauchy dispersion model was used to determine both the combined refractive index and thickness, before and after the incorporation of GOx. Then the volume fractions of the buffer molecules, the polymer and the enzyme were determined by a two- and three-component Bruggemann-EMA model, respectively, using fixed values of thickness as determined from the Cauchy modeling. Here, for the polymer, the fixed index of refraction of the dry polymers and for the enzyme the refractive index of human serum albumin was used with n(631.5) = 1.578 (Arwin, 1986). For the optical properties of the ambient, the refractive index *n*(λ) was measured with a digital multiple wavelength refractometer (DSR-lambda, Schmidt+Haensch GmbH& Co.) at eight different wavelengths from 435.8 to 706.5 nm. The amount of enzyme incorporated into the polymer brush layer was calculated according to a modified De-Feijter approach (De Feijter et al., 1978; Bittrich, 2010).

### 2.4. QCM-D Modeling

Shifts in frequency and dissipation of the odd overtones (j = 3, 5, 7, 9, 11) were modeled using a Voigt-Voinova approach for one homogeneous viscoelastic layer with a fixed density of 1 g cm^−3^ (Voinova et al., 1999). Measurements were referenced to the measurement with the smallest dissipation value. The software QTools (Biolin Scientific, Sweden) was used for modeling.

### 2.5. Attenuated Total Reflection-Fourier Transform Infrared Spectroscopy

Attenuated total reflection - Fourier transform infrared (ATR-FTIR) spectra were recorded with an IFS55 spectrometer (Bruker Optics GmbH, Leipzig, Germany) using the “single-beam-sample-reference” (SBSR) method (OPTISPEC, Zürich, Switzerland) (Fringeli, 1992). A special cell-design, appropriate for the SBSR-method was used (Fringeli, 1992; Müller et al., 1999). The SBSR concept implies the recording of single-channel spectra *I*_S, R_(ν) separately on the sample (*S*) and the reference (*R*) half of the ATR crystal. Normalizing the single-channel spectra according to *A*(ν) = −log(*I*_*S*_(ν)/*I*_*R*_(ν)) results in absorbance spectra (*A*(ν)) with a proper compensation of background absorption, due to the SiO_*x*_ layer, solvent, water vapor, and ice on the detector window.

## 3. Results and Discussion

EDC/NHS-Activation was used to covalently immobilize GOx to PAA Guiselin brushes, via reaction of amine groups on the surface of the enzyme to the carboxylic groups of the polymer. Since the study of physical adsorption of GOx to PAA brushes showed that almost no unspecific adsorption occurs at pH 6, this pH value was chosen for the coupling reaction (Koenig et al., 2016). The course of experiments was as follows: (I) rinsing with sodium phosphate buffer solution (*c*(Na^+^)=0.01 M) at pH 7.4, (II) rinsing with buffer solution at pH 6, (III) rinsing with a solution of EDC and NHS in pH 6, (IV) reaction with a solution of GOx in pH 6 under stagnant conditions, interrupted by short rinsing steps to allow a fresh enzyme solution to enter the measurement cell, (V) rinsing with buffer solution at pH 6, (VI) rinsing with buffer solution at pH 7.4, (VII) rinsing with buffer solution at pH 6.

**Figure 1:**
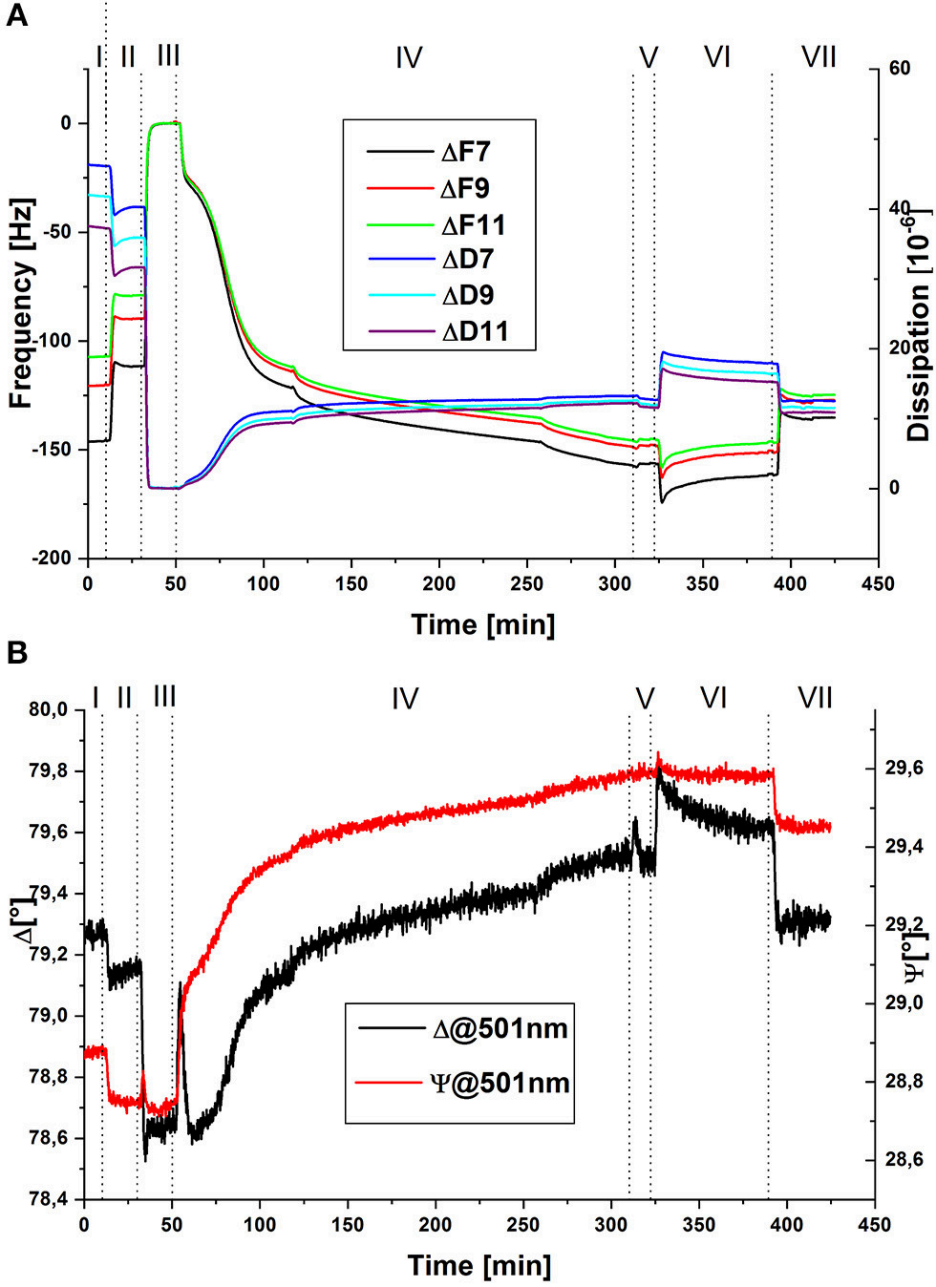
Exemplary raw data of QCM-D for three different overtones **(A)** and SE for one wavelength **(B)** of *in-situ* measurements during the covalent immobilization of GOx in PAA brushes; (I) pH 7.4, (II) pH 6, (III) EDC/NHS in pH 6, (IV) GOx in pH 6, (V) pH 6, (VI) pH 7.4, (VII) pH 6; QCM-D data are shown in reference to the minimum value at around 40 min.

**Figure 2:**
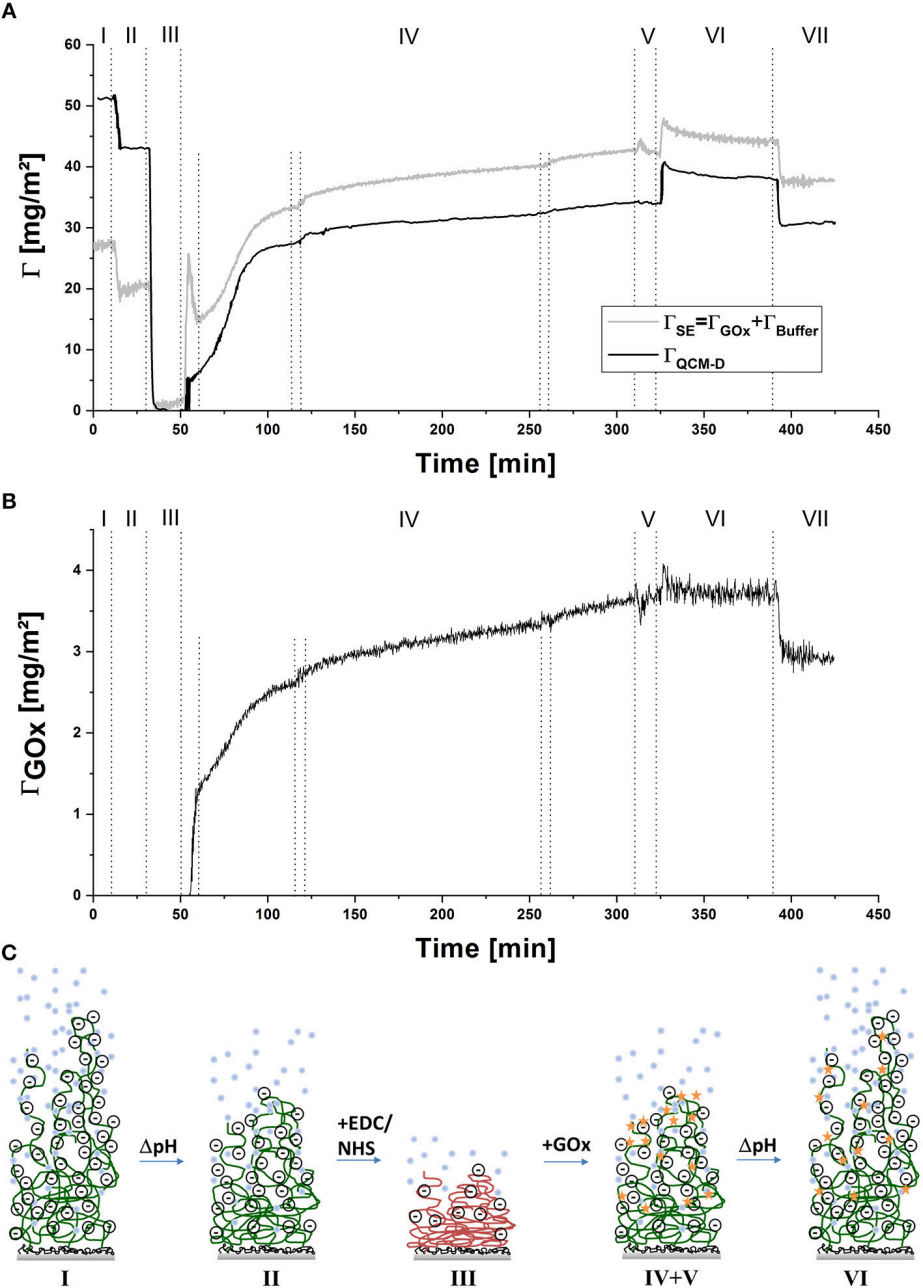
**(A)** Comparison of the change in areal mass determined from QCM-D and SE during the covalent immobilization of GOx in PAA brushes, **(B)** Change in Γ_*GOx*_ during covalent coupling to PAA brushes determined from SE measurements, **(C)** scheme of the proposed changes in conformation and hydration of the polymer brush layer during the coupling process; (I) pH 7.4, (II) pH 6, (III) EDC/NHS in pH 6, (IV) GOx in pH 6, (V) pH 6, (VI) pH 7.4, (VII) pH 6; data are shown in reference to the minimum value at around 40 min.

To follow the process *in-situ*, combined QCM-D/SE measurements were conducted. Exemplary raw data of the change in frequency and dissipation of three overtones and of the ellipsometric angles at one wavelength are displayed in Figure 1. Data are shown in reference to the minimum value at around 40 min. The dotted lines indicate the times of switching valves or, in the case of the shorter lines in step IV, switching the peristaltic pump on and off. For quantitative evaluation, data was modeled by a Voigt-Voinova approach (QCM-D) and an EMA approach, with a modified De-Feijter approach (SE). Figure 2A displays best-match modeling results for the change in areal mass (Γ), detected by the two techniques. For comparison with QCM-D data, which displays only the relative change in mass of molecules adsorbing and reacting the polymer layer, the SE curve displays the combined mass of buffer and enzyme molecules in the layer without the polymer mass.

**Figure 3:**
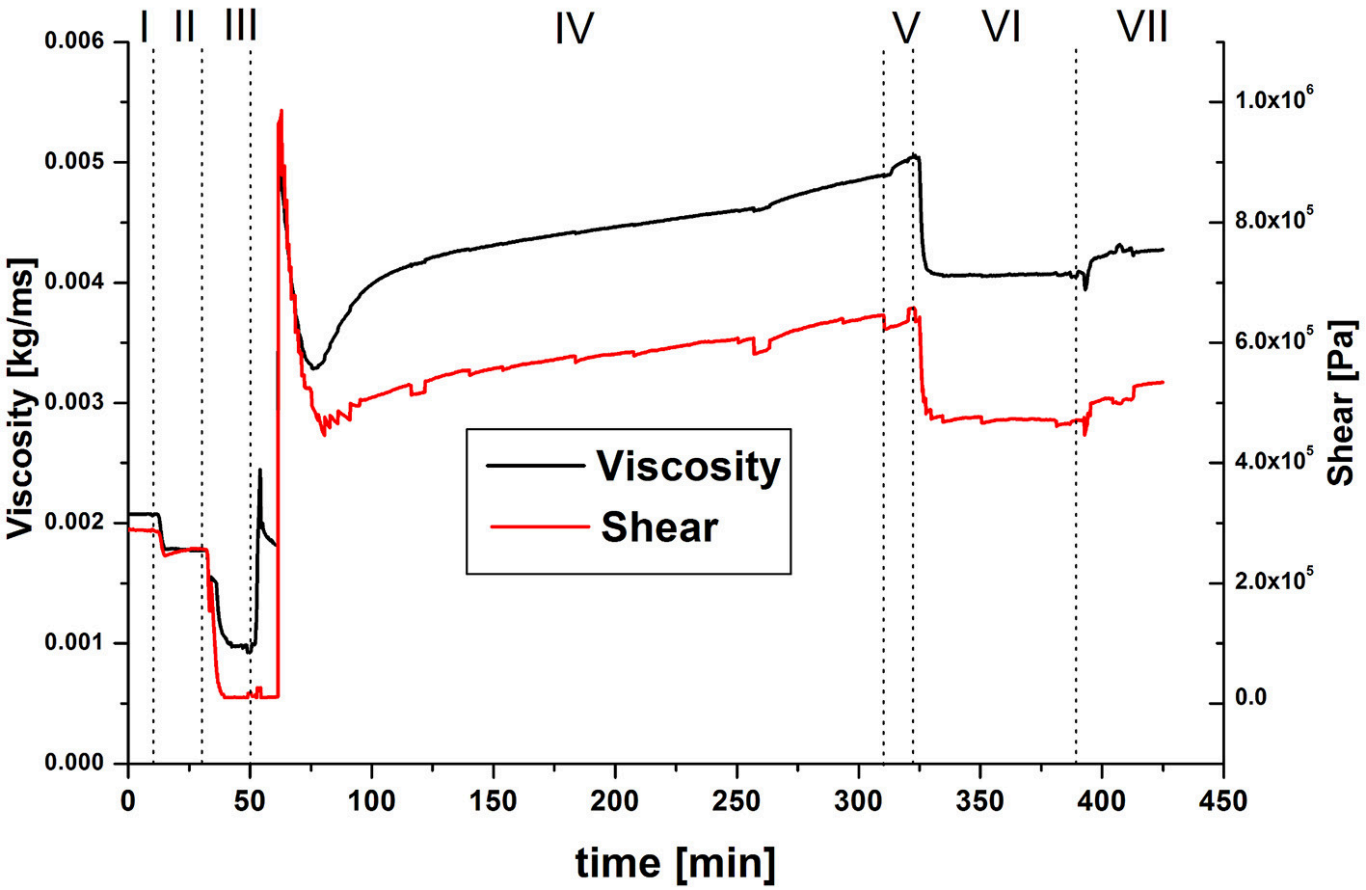
QCM model data of the change in viscoelasticity during the chemical coupling of GOx to PAA; (I) pH7.4, (II) pH6, (III) EDC/NHS in pH6, (IV) GOx in pH6, (V) pH6, (VI) pH7.4, (VII) pH6.

A loss of mass is detected in the transition from step I to step II. Here, lowering of pH causes protonation of carboxylate groups, accompanied by the loss of water molecules and counter ions. Upon addition of EDC/NHS (step III), the negatively charged carboxylic groups react first with EDC then further with NHS to form a neutral succinimidyl-ester intermediate, which causes further deswelling, again detected as a loss of mass. QCM-D raw data was referenced to this step, since dissipation values were the lowest at this point of the measurement. For comparison, an offset to SE modeled data was applied as well—originally a remaining areal mass of ~7 mg m^−2^ of buffer and a thickness of ~10 nm had been calculated, indicating that the polymer layer is almost completely collapsed. The addition of enzyme solution in step IV leads to two simultaneous reactions, which are both accompanied by an increase in areal mass: on the one hand, GOx molecules adsorb to, and subsequently react with the activated polymer layer. On the other hand, hydrolysis of the succinimidyl-ester occurs, followed by the re-charging and penetration of solvent molecules into the polymer layer. This step was done under overall non-flowing conditions, to increase the contact time of the enzyme with the activated polymer layer, allowing the coupling reaction to take place. While the pump is still running during the initial 10 min of introduction of GOx, an overshoot of Γ_*SE*_ is detected (reflected also in the Psi and Delta values (Figure 1), followed by a slower decrease, which is only stopped by switching the pump off. In the values of Γ_*QCM*_ and in QCM-D raw data, this trend is not detected, while viscosity and shear modulus (see Figure 3) display a similar overshoot behavior, with an even longer recovery time. Intermittent rinsing with enzyme solution does not cause this overshooting again, indicating that it is caused by the initial rearrangement of polymer chains and the electrical double-layer. However, the introduction of fresh enzyme solution into the cell leads to a slightly increased adsorption rate, since the depleted volume over the sample surface is renewed. After approximately 5 h, the reaction was aborted, as the rate of adsorption almost reaches zero. Rinsing with buffer solution at pH 6 (step V), does not change the areal mass, indicating that no loosely attached enzymes are present on the surface. To remove physically adsorbed GOx, the sample was rinsed with buffer solution at pH 7.4 (step VI). At first, a rapid increase in mass was detected, caused by swelling of the PAA layer, followed by a slower decrease, indicating the removal of some GOx molecules or slow release of counter ions or buffer molecules from the layer. The last rinsing step with buffer solution at pH 6 (step VII), again caused the partial de-swelling of the polymer layer. Interestingly, the areal mass detected by QCM-D after chemical coupling of GOx was less, compared to the original value of the PAA brush swollen in buffer solution at pH 6. Contrary to this, in SE modeled data, an overall increase of ~11 mg m^−2^ by the incorporation of solvated enzyme was detected. This contradictory behavior is also mirrored in the raw data (see Figure 1). One explanation would be a decrease in the amount of water acoustically coupled to the polymer layer, which cannot be detected by SE due to the insufficient optical contrast (Bittrich et al., 2010; Rodenhausen and Schubert, 2011; Adam et al., 2017): interaction with the enzyme leads to a decreased interaction of the polymer layer with the ambient, caused by partial neutralization of charges and polar groups.

The finding in step III, that the polymer brush collapses upon activation with EDC/NHS, is very interesting since the conformation of the polymer layer has an impact on the location of the immobilized enzyme: at the surface of this compact, collapsed polymer layer, chain segments of almost the whole polymer length are randomly exposed to the ambient solution. This means that the enzyme does not have to diffuse through the polymer layer in order to react with segments located closer to the grafted end of the polymer chains, and the immobilized enzyme will be distributed through the whole polymer brush when the chains are swollen again. On the other hand, the amount of enzyme that can be bound to the polymer brush is limited, since only the chain segments on the surface of the collapsed brush layer are accessible and not the whole volume of the swollen brush.

Figure 2B displays the change in the amount of GOx, modeled from SE data using the refractive index for dry protein and a modified De-Feijter approach (De Feijter et al., 1978; Bittrich, 2010). Upon introduction of the enzyme in step IV, a continuous increase of GOx can be observed. This increase is considerably slower than the increase of Γ_*SE*_, again pointing to the fact that the overshoot detected in Γ_*SE*_ is caused by a change in buffer molecules included in the optical box model. During rinsing with buffer of pH 6 and pH 7.4 (steps V + VI), no change in Γ_*GOx*_ is suggested by the model. By changing the pH of the ambient solution back to pH 6 (step VII) an unexpected, step-like release of enzyme is observed. Together with the slow decrease in Γ_*SE*_, detected upon rinsing with buffer at pH 7.4, this points to the explanation that by increasing the pH in step VI, the charge of the polymer and the adsorbed enzyme changes, concomitant with re-arrangement of the non-covalent bonds between enzyme and polymer and the slow release of buffer molecules and counter ions from the layer, but no complete rupture of hydrogen or other physical bonds. Upon decrease of the pH in step VII and deswelling of the polymer chains, the now more loosely adsorbed enzyme molecules desorbed at once. Overall, a final amount of ~3 mg m^−2^ GOx was calculated to be immobilized in the PAA brush layer. This is considerably more than the amount found after physical adsorption of GOx on PAA brushes at similar pH conditions (~0.7 mg m^−2^)(Koenig et al., 2016). Figure 2C displays a schematic of the proposed changes in conformation and hydration of the polymer brush layer during the coupling process.

Both the viscosity and the shear modulus (Figure 3) display the expected behavior during the process of covalent coupling of GOx to PAA, at first: as the polymer layer deswells (steps I–III), the viscoelasticity decreases as well, after a small overshoot, since the layer becomes more rigid. Upon incorporation of enzyme, the viscoelasticity increases again. Contrary to steps I + II, upon increasing the pH in step VI, the viscoelasticity decreases, although the change in areal mass suggests swelling of the polymer layer. When the pH is lowered again in step VII, no full recovery of the values of step V is detected, indicating that the decrease in viscoelasticity is caused by an irreversible process. When comparing the swollen polymer brush surface in pH 6 with and without an immobilized enzyme, an overall increase of viscoelasticity is detected.

**Figure 4:**
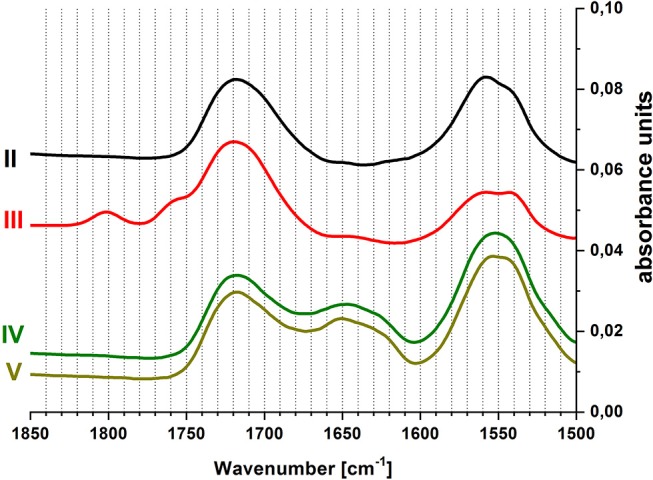
*In-situ* ATR-FTIR spectra of covalent coupling of GOx to PAA brushes, (II) pH 6, (III) EDC/NHS in pH 6, (IV) GOx in pH 6, (V) pH 6; curves were shifted along the vertical axis for better display.

To confirm the chemical reactions occurring on the surface, ATR-FTIR spectra were recorded *in-situ* during this process. Figure 4 displays measurements at the end of each step of the coupling process. In the range of 1,500–1,900 cm^−1^ characteristic vibrational bands of the carbonyl group can be found. The PAA brush layer shows two vibrational bands in this region: the peak at 1,550 cm^−1^ is assigned to carboxylate groups, while the peak at 1,720 cm^−1^ is caused by the protonated carboxylic acid (Müller, 2002). Upon activation with EDC/NHS (step III), two additional bands appear at 1,760 and 1,800 cm^−1^. These can be assigned to the vibrations of the carbonyl groups in the succinimidyl ring (Frey and Corn, 1996). At the same time, the intensity of the carboxylate vibrational band decreases, due to the reaction of these groups with EDC/NHS. After the coupling of GOx (step IV), the succinimidyl vibrations vanish. A signal at 1,650 cm^−1^, along with the increase of the signal at 1,550 cm^−1^, indicates the presence of amide bonds and proves the immobilization of enzyme on the surface. Rinsing with buffer at pH 6 (step V) does not change the spectrum.

To ensure the preservation of the enzymatic activity of GOx covalently immobilized on PAA brushes, the activity was tested by a colorimetric assay. These results are published in a separate manuscript and discussed in comparison with the activity of the free enzyme in solution and physically adsorbed to PAA, as well as other types of polyelectrolyte brushes (Ferrand-Drake del Castillo et al., 2019). The immobilized enzyme shows satisfying activity, although the specific activity is about four times less than in the solution. This is probably due to the active site being less readily available on the immobilized enzyme than on the free enzyme. Interestingly, the covalently bound enzyme is less inhibited than the small amount of physically adsorbed enzyme.

**Figure 5:**
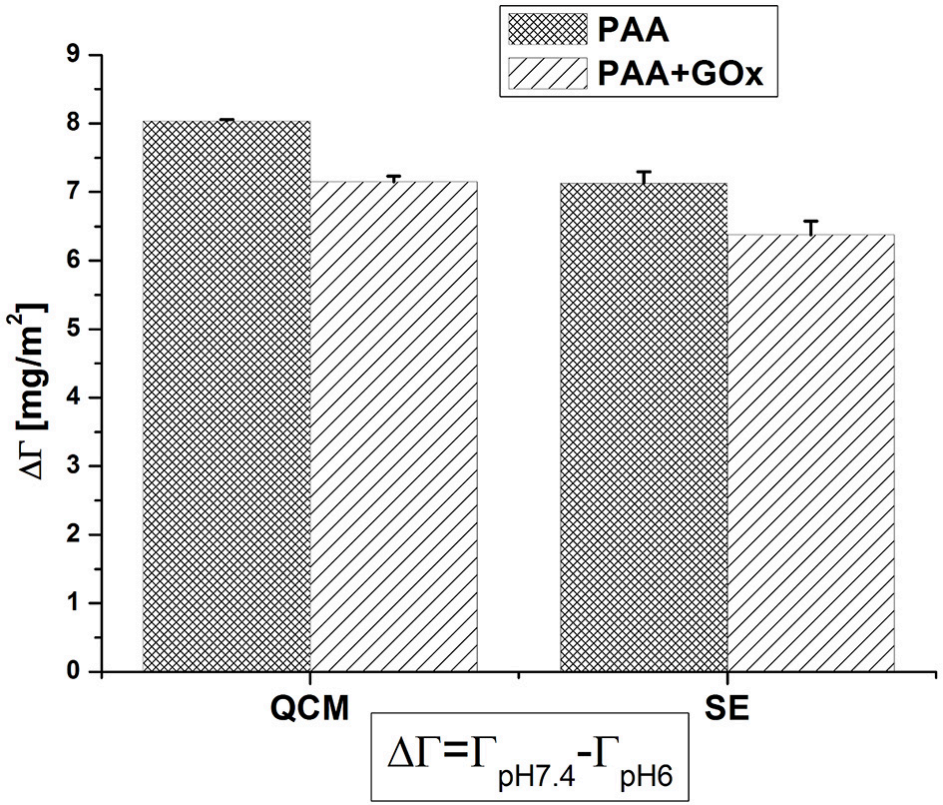
Comparison of the pH responsive behavior of PAA before and after immobilization of GOx; the plot displays the difference between the areal mass at pH 7.4 and at pH 6.

For possible future applications, the pH responsive behavior of PAA brushes with immobilized GOx molecules is of importance. Therefore, the difference ΔΓ in between pH 7.4 and pH 6 was calculated. Figure 5 compares ΔΓ before and after the immobilization of enzyme, as detected by the two techniques QCM-D and SE. A slight decrease of ~10% of the original value, after the immobilization of GOx, was noticed. This can be related to some of the carboxyl groups being involved in the covalent bond to the enzymes and not free for protonation anymore. Comparing both characterization techniques, the degree of switching is always slightly higher when detected by QCM-D than by SE, since QCM-D is more sensitive to solvent molecules acoustically coupled to the polymer layer and the diluted outer region of the brush (Adam et al., 2017).

## 4. Conclusions

In summary, the immobilization of enzymes in polymer brushes can be comprehensively investigated *in-situ* using the combined setup of QCM-D together with SE. Both the conformational state of the polymer layer and the incorporation of enzyme molecules can be monitored directly during the reaction process. Carbodiimide chemistry was used to covalently couple the model enzyme GOx to PAA Guiselin brushes. The activation of the carboxylic acid groups by esterification with EDC/NHS leads to the almost complete collapse of the polymer chains. Upon introduction of the enzyme solution, the brush becomes re-hydrated which is detected as an increase of buffer molecules in the polymer layer. Simultaneously, enzymes are incorporated into the polymer brush via a reaction with the amine groups on the surface of the enzyme. The final amount of immobilized enzyme is significantly higher than the amount after physical adsorption under similar pH conditions. Comparing the pH-sensitive swelling behavior of the brushes with and without immobilized enzyme, the swelling ratio is only slightly reduced by the presence of the enzymes, which is promising for the future application in stimuli-responsive biocatalytic devices and biosensor systems.

## Data Availability

The raw data supporting the conclusions of this manuscript will be made available by the authors, without undue reservation, to any qualified researcher.

## Author Contributions

MK performed the experiments, analyzed the data, and wrote the manuscript. MM and K-JE aided in the data analysis. MK, UK, MS, and PU designed the experiments. All authors read and approved the manuscript.

### Conflict of Interest Statement

The authors declare that the research was conducted in the absence of any commercial or financial relationships that could be construed as a potential conflict of interest.
